# The impact of age and gut microbiota on Th17 and Tfh cells in K/BxN autoimmune arthritis

**DOI:** 10.1186/s13075-017-1398-6

**Published:** 2017-08-15

**Authors:** Fei Teng, Krysta M. Felix, C. Pierce Bradley, Debdut Naskar, Heqing Ma, Walid A. Raslan, Hsin-Jung Joyce Wu

**Affiliations:** 1 Department of Immunobiology, University of Arizona, Tucson, AZ 85719 USA; 2 Arizona Arthritis Center, College of Medicine, University of Arizona, Tucson, AZ 85719 USA

**Keywords:** Rheumatoid arthritis, Age, Gut microbiota, Tfh, Th17, Animal model, Lung pathology

## Abstract

**Background:**

Age is an important risk factor for rheumatoid arthritis (RA), which often develops in middle age. However, how age-associated changes in immunity impact RA is poorly understood. Gut microbiota are known to be involved in the pathogenesis of RA, but the effects of microbiota in older subjects remain mostly unknown.

**Methods:**

We used segmented filamentous bacteria (SFB), a gut commensal species with immunomodulatory effects, and K/BxN mice, a T cell receptor (TCR) transgenic model, to study the effect of age and microbiota on autoimmune arthritis. Comparing young and middle-aged K/BxN T cells of the same TCR specificity allows us to study T cells with an age focus eliminating a key variable: TCR repertoire alteration with age. In addition to joints, we also studied pathological changes in the lung, an important extra-articular RA manifestation. We used flow cytometry to evaluate T follicular helper (Tfh) and T helper 17 (Th17) cells, as they both contribute to autoantibody production, a key disease index in both RA and K/BxN arthritis.

**Results:**

Middle-aged K/BxN mice had aggravated arthritis and pathological changes in the lung compared to young mice. Middle-aged mice displayed a strong accumulation of Tfh but not Th17 cells, and had defective Th17 differentiation and low expression of interleukin-23, a critical cytokine for Th17 maintenance. Although a soaring Tfh cell population accompanied by robust germinal center B cell responses were found in middle-aged mice, there was decreased cycling of Tfh cells, and SFB only induced the non-Tfh cells to upregulate Bcl-6, the Tfh master transcription factor, in the young but not the middle-aged group. Finally, the accumulated Tfh cells in middle-aged mice had an effector phenotype (CD62L^lo^CD44^hi^).

**Conclusion:**

Age-dependent Tfh cell accumulation may play a crucial role in the increased autoimmune disease phenotype in middle-age. SFB, a potent stimulus for inducing Tfh differentiation, fails to promote Tfh differentiation in middle-aged K/BxN mice, suggesting that most of the middle-aged Tfh cells with an effector phenotype are Tfh effector memory cells induced at an earlier age. Our results also indicate that exposure to immunomodulatory commensals may allow the young host to develop an overactive immune system reminiscent of that found in the middle-aged host.

## Background

Age-related reductions in immune functions in vaccination, infection, and cancer have been better characterized [1]; however, little is known regarding the age-associated changes in immune function that result in autoimmunity. This is a pivotal field that warrants a strong research focus as there is a clear association between age and increased incidence of many autoimmune diseases including rheumatoid arthritis (RA), myositis, and Sjögren’s syndrome [2]. RA onset can occur at any age, but it usually develops in middle-aged adults between 40 and 60 years old [3]. Recently, gut microbiota have been demonstrated to have a profound influence on host health and disease [4–7]. Reports have found that microbiota in older people are different from those in younger adults [8, 9]. We and others have demonstrated that gut microbiota can act as an environmental cue to induce autoimmune arthritis in both humans and mice [10–13]. However, a causative effect of microbiota in age-related disease development among older individuals remains to be determined.

Here, we aimed to investigate the age-associated and microbiota-associated impacts on autoimmune disease. Because RA is an autoantibody-mediated autoimmune disease [14, 15], we are interested in two crucial subsets of effector T cells that provide help to B cells for autoantibody production. T follicular helper (Tfh) cells are a crucial subset of CD4^+^ T cells that helps B cells produce high-affinity and high-titer antibodies [16–18], and an excessive Tfh cell response can lead to many autoimmune conditions including RA [19]. T helper 17 (Th17) cells, a T effector cell type involved in many autoimmune diseases, promote both autoantibody production and inflammation [20]. We used a TCR transgenic (Tg) autoimmune arthritis model, K/BxN mice [21], to study the effect of age and microbiota on autoimmune Tfh and Th17 cells. K/BxN mice are an autoimmune arthritis model in which transgenic KRN T cells recognize glucose-6-phosphate isomerase (GPI), the self-antigen (Ag) presented by major histocompatibility complex (MHC) class II I-A^g7^ molecules. As in humans with RA, autoantibodies are crucial for disease pathogenesis in K/BxN mice [21]. By examining TCR Tg T cells in young and middle-aged K/BxN mice, we can compare T cells of the same TCR specificity with the only difference being the age of the mouse. This has been shown to be beneficial in aging T cell studies as it eliminates change in the T cell repertoire, an important variable that complicates the interpretation of aging T cell function [22].

In contrast to the abundant gut-luminal commensals, mucosa-associated commensal species such as segmented filamentous bacteria (SFB) represent a minor but important part of the commensal community, as they can powerfully modulate host immunity [23–27]. Our previous studies have shown that SFB-induced Tfh and Th17 cells contribute significantly to autoantibody production in young K/BxN mice, and a lack of either T effector cell type strongly ameliorates autoantibody production and autoimmune arthritis development [11, 12]. Here we reveal that despite both Tfh and Th17 cells having been reported to participate in the pathogenesis of autoimmune arthritis in most of the studies using young adult mice in experimental settings, there is a clearly age-associated accumulation of Tfh but not Th17 cells in the middle-aged group compared to their young counterparts. Our results suggest that most of the accumulated middle-aged Tfh cells are of the effector phenotype. Our results further indicate that exposure to commensal bacteria SFB causes the young host to develop an overactive immune system, with a strong elevation in their Tfh cell response reminiscent of the middle-aged condition.

## Methods

### Mice

K/BxN mice were generated by crossing KRN TCR transgenic mice on the C57BL/6 (B6) background with NOD mice (F1 mice of KRN/B6 x NOD). Ankle thickness was measured with a caliper (J15 Blet micrometer) as described previously [11]. All mice were housed at the SPF animal facility at the University of Arizona. The young mice were used at 6–8 weeks of age, while the middle-aged K/BxN mice were used at 10–15 months of age using the age guideline of the Jackson Laboratory (Jax) described as:

Middle age refers to a phase during which senescent changes can be detected in some, but not all, biomarkers of aging. For the middle-aged group, mice should be at least 10 months old. Senescence processes that begin in younger adults (for example, collagen cross linking and accumulation of activated/memory T cells) often can be detected by then. The upper age limit for the middle-aged group is typically 14–15 months, because at this age, most biomarkers still have not changed to their full extent, and some have not yet started changing.

All experiments were conducted according to the guidelines of the Institutional Animal Care and Use Committee at the University of Arizona under the protocol reference number 11-278.

### Preparation of single-cell suspension from the lung and small intestine-lamina propria (SI-LP)

Lungs were perfused with 10 ml PBS to remove blood, and were finely minced. Minced lung was placed into 10 ml of digestion buffer containing 1 mg/ml each of Collagenase D (Roche) and MgCl_2_ and 0.15 mg/ml DNase I (Sigma) in DMEM (HyClone). Lungs were digested for 20–25 min at 37 °C at 200 rpm then passed through a 100-μm cell strainer. A plunger from a 5-ml syringe was then used to grind remaining tissue pieces through the cell strainer. SI-LP cells were isolated as described, with some modification [11]. Briefly, Peyer’s patches (PPs) were removed from the small intestine. The small intestine was opened longitudinally and excess mucus was removed by scraping gently with forceps along the length of the intestine. The intestine was then thoroughly washed in 5 mM ice-cold ethylenediaminetetraacetic acid (EDTA) in PBS and cut into 1-cm pieces, which were incubated in 40 ml of 5 mM EDTA and 0.145 mg/ml of DL-dithiothreitol (DTT) in DMEM for 60 min at 37 °C horizontally at a rotation speed of 100 rpm. After incubation, the epithelial cell layer, containing the intraepithelial lymphocytes, was removed by shaking, and then cleaned by pressing intestinal pieces over a 100-μm nylon layer on top of a paper towel. Intestinal pieces were then transferred to an eppendorf tube with 600 μl of digestion solution containing 1 mg/ml Collagenase D (Roche), 0.15 mg/ml DNase I (Sigma), and 200 ng/ml liberase Cl (Roche). The pieces of intestine were finely minced and transferred to a 50-ml conical tube containing 5 ml of digestion solution. Digestion was performed by incubating the pieces at 37 °C for 15 min with rotation at 200 rpm. After digestion, 10 ml EDTA/PBS was added and the solution was passed first through a 100-μm cell strainer, then through a 40-μm cell strainer. Cells were centrifuged and washed again with EDTA/PBS before being resuspended in 10% FBS DMEM for stimulation.

### Antibodies and flow cytometry

For surface staining, fluorophore-conjugated monoclonal antibodies (mAbs) specific for CD4 (RM4-5), CD19 (6D5), CD45 (30-F11), PD-1 (RMP1-30), CD11b (M1/70), CD44 (IM7), CD62L (MEL-14), and TCRβ (H57-597) were obtained from BioLegend. Abs recognizing Fas (Jo2), CXCR5 (2G8), and TCR Vβ14 (14-2) were from BD Pharmingen. Anti TCR Vβ6 (RR4-7) was from eBioscience. FITC-conjugated peanut agglutinin (PNA) was from Vector Laboratories. For intracellular cytokine staining, cells were incubated for 4 h with BD GolgiPlug (1:1000 dilution), 50 ng/ml phorbol 12-myristate 13-acetate, and 1 μM ionomycin in DMEM (HyClone) supplemented with 10% FCS, 1% nonessential amino acids, penicillin, streptomycin, and glutamine at 37 °C. Intracellular cytokine staining was performed with Cytofix/Cytoperm (BD Pharmingen). Abs recognizing interleukin-17A (IL-17A, TC11-18H10.1) were obtained from BioLegend and Abs recognizing IL-23 (fc23cpg) were obtained from eBioscience. For intra-nuclear staining, buffers from a Foxp3 Staining Buffer Set (eBioscience) were used to stain Abs recognizing Ki-67 (B56, eBioscience) and Bcl-6 (K112-91, BD Pharmingen). Cells were run on an LSRII (BD Biosciences), and analyses were performed using FlowJo (TreeStar) software.

### Microbiota reconstitution and quantification

Our SPF mouse colony and SFB colonization were maintained as described previously [12]. Briefly, our SPF mouse colony was originally derived from Jax and was verified as SFB-negative (SFB– hereafter). SFB were initially introduced to our mouse colony by gavaging mice with feces containing SFB, from Taconic B6 mice. Later, SFB were passed by gavaging mice with feces containing SFB, collected from the SFB+ mice housed in our colony. SFB– mice were weaned at 21 days old and rested for 1 day. Then, mice were orally gavaged with feces containing SFB, collected in-house for 3 consecutive days starting at 23 days old. The middle-aged K/BxN mice aged 10–15 months were gavaged at the same time as the young mice. The SFB– mice were the un-gavaged littermate controls. Unless otherwise mentioned, the SFB colonization status was examined on day 10 after SFB gavage by SFB-specific 16S rRNA quantitative PCR as previously described [11].

### ELISA

Anti-GPI Ab titers were measured as described [11]. Briefly, ELISA plates were coated with recombinant mouse GPI at 5 μg/ml, and diluted mouse serum was added. Subsequently, plates were washed and alkaline-phosphatase (AP)-conjugated anti-mouse IgG Ab was added. After the final wash, AP substrate was added and titers were quantified as optical density values using an ELISA reader. The Ab titers were expressed as arbitrary units, which were calculated from serial dilutions of sample serum and defined as the reciprocal of the highest dilution that gave a background optical density (OD) value set as 0.15.

### In vitro Th17 polarization

Splenic naïve CD4^+^ T cells (2.5 × 10^4^) from SFB young or middle-aged K/BxN mice were enriched by fluorescence-activated cell sorting (FACS Aria) and in vitro cultured in 96-well plates for 4 days in Th17 polarization conditions: anti-CD3ε (plate-coated, 2 μg/ml), anti-CD28 (2 μg/ml), anti-IL-2 (10 μg/ml, JES6-1A12, BioXCell), IL-6 (50 ng/ml, PeproTech), transforming growth factor (TGF)β1 (1 ng/ml, PeproTech), 6-formylindolo [3,2-b] carbazole (FICZ), (300 nM, Enzo Life Sciences).

### Immunohistochemical analysis

Lung sections from SFB– and SFB+ K/BxN mice were perfused and fixed with 10% (vol/vol) buffered formalin and stained with hematoxylin and eosin (H&E) for histological evaluation of lymphocyte aggregation. Image analysis was performed using ImageJ software (NIH, Bethesda, MD, USA).

### Statistical analysis

Differences were considered significant with *p* < 0.05 analyzed by Student’s *t* test (two-tailed, unpaired) or two-way analysis of variance (ANOVA) (Prism 6, Graph-Pad Software), with significance level denoted as: *p < 0.05, **p < 0.01, ***p < 0.001, and ****p < 0.0001. We also used Prism to calculate Spearman correlation.

## Results

### The impact of age and gut microbiota on RA-related autoimmune arthritis and pathological changes in the lung

**Fig. 1:**
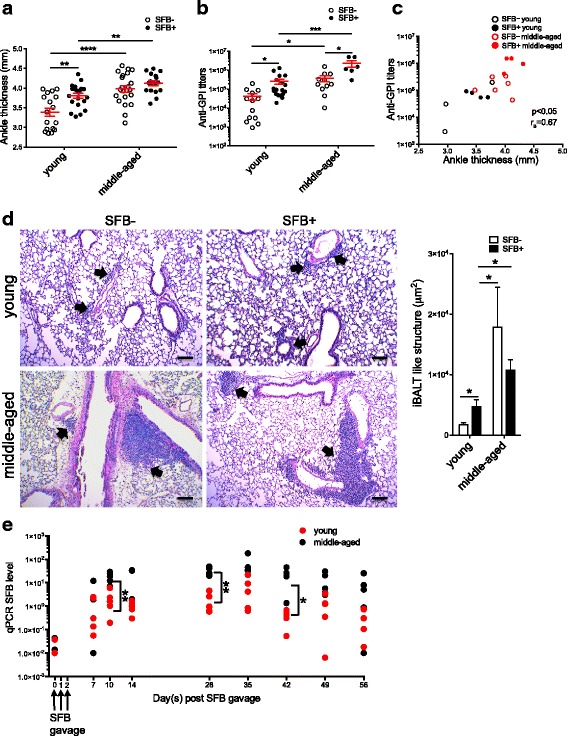
Age and gut microbiota enhance rheumatoid arthritis related autoimmune arthritis and pathological changes in the lung. **a** K/BxN mice aged 23 days (*young*) or 10–15 months old (*middle-aged*) were gavaged for 3 consecutive days with segmented filamentous bacteria (*SFB*) or left non-gavaged. At 20 days after the first gavage, ankle thickness was measured and is shown as mean ± SEM. Non-arthritic adult mice have a basal ankle thickness of ~2.8–2.9 mm (male mice have slightly greater ankle thickness than female mice) and thus ankle thicknesses of >3 mm are considered as indicative of arthritis (each *dot* indicates the mean value of the ankle thickness from both ankles of the same mouse). **b** Serum from young and middle-aged K/BxN mice was collected 20 days after the first SFB gavage. Anti-glucose-6-phosphate isomerase (*Anti-GPI*) autoantibody titers were determined by ELISA and are shown as mean ± SEM. **c** Spearman correlation between anti-GPI autoantibody titers and ankle thickness in SFB– and SFB+ young or middle-aged K/BxN mice (total of 17 mice from three independent experiments). **d** Representative images of lung histologic staining (H&E) from non-gavaged and day-20 SFB-gavaged young and middle-aged K/BxN mice. The combined and quantified data on areas of inducible bronchus-associated lymphoid tissue (*iBALT*)-like structures from 10 random, non-overlapped fields of each image are also shown (n = 3–8 mice in each group). **e** The SFB colonization levels of young and middle-aged K/BxN mice from the experiments as shown in **a** were checked at the indicated time points after SFB gavage; *days* indicates the number of days post first SFB gavage

Autoantibodies are a hallmark of B-cell-mediated autoimmune diseases, including RA. As in patients with RA, serum autoantibodies serve as the disease index in the K/BxN model and indeed, passive transfer of K/BxN arthritic serum (containing anti-GPI autoantibodies) into wild-type mice is sufficient to induce arthritis development [21]. Thus, to examine the effects of microbiota and age on autoimmune arthritis development, we first compared the ankle thickness and anti-GPI autoantibody titers between young and middle-aged K/BxN mice. As reported previously, SFB colonization significantly enhanced arthritis development in the control young K/BxN mice as indicated by the increase in ankle thickness (Fig. 1a). The majority (~65%) of SFB– mice developed arthritis (ankle thickness >3 mm) at a young age, ~6 weeks old, and the rest (~35%) developed arthritis between 6 weeks and 10 months, by middle age. This is in contrast to the SFB+ group, in which all mice developed arthritis at a young age. This disease phenotype corresponded with an increase in anti-GPI autoantibody titers (Fig. 1b). In the middle-aged group, severe arthritis had already been observed in SFB– mice, and SFB colonization did not further augment arthritis severity (Fig. 1a) and only mildly increased the anti-GPI titers (Fig. 1b). Overall, we found that middle-aged K/BxN mice displayed greater ankle thickness and anti-GPI titers compared to their young counterparts.

Next, we examined whether there was a correlation between anti-GPI titer and ankle thickness in K/BxN mice. Specifically, we pooled all mice from three independent experiments for which we have recorded data containing ankle thickness for each mouse and its corresponding anti-GPI titer, and used Prism to compute the *p* value for nonparametric (Spearman) correlation. Our data indicate there is significant and strong correlation between autoantibody titer and ankle thickness (Fig. 1c). Inducible bronchus-associated lymphoid tissue (iBALT) is a type of ectopic lymphoid tissue found in the lungs of patients with RA and is positively correlated with the severity of the patient’s lung disease [28]. Previously we have demonstrated that SFB colonization provoked young K/BxN mice to develop iBALT-like structures closely resembling the iBALT formations in patients with RA [29, 30]. Here, we compared iBALT lesions between young and middle-aged groups with or without SFB colonization. SFB induced iBALT areas in young K/BxN mice. In contrast, middle-aged K/BxN mice displayed strong iBALT lesions compared to young mice regardless of SFB status (Fig. 1d). Next, we evaluated the ability of SFB to colonize young and middle-aged K/BxN mice and found that SFB was able to colonize and persist in middle-aged hosts at a higher level than in young hosts at several time points (Fig. 1e). However, the difference between the middle-aged and young groups seemed to subside by day 49 after gavage.

### SFB-induced Th17 response is impaired in the middle-aged group

**Fig. 2:**
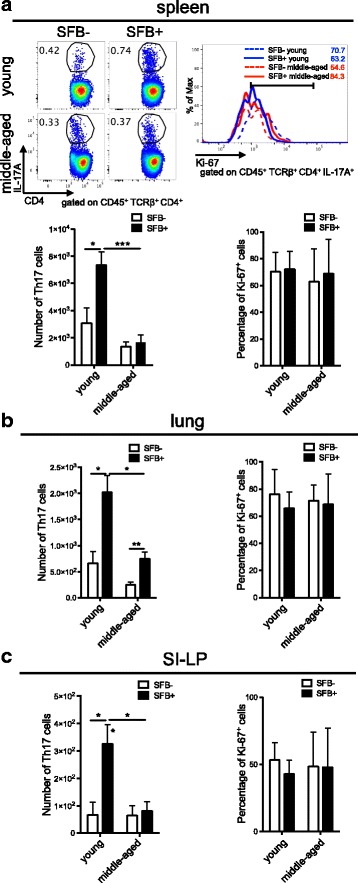
Reduced SFB-mediated Th17 induction in middle-aged compared to young K/BxN mice. (**a**) Representative plots of Th17 cells and histogram overlays of Ki-67^+^ Th17 cells in spleen of SFB– and SFB+ young or middle-aged K/BxN mice are shown, along with the quantitative data of Th17 cell numbers and percentage of Ki-67^+^ Th17 cells (n=4-5 mice in each group). **b** and **c**. The quantitative data of Th17 cell numbers and percentage of Ki-67^+^ Th17 cells in lung (**b**) and SI-LP (**c**) from experiments in 2A are also shown

Because Th17 cells have been reported to be involved in the pathogenesis of autoimmune diseases, including in the K/BxN model, we first compared whether there is an elevated number of Th17 cells in the spleen of middle-aged mice. In young mice, SFB is known as a strong Th17 inducer and SFB-induced Th17 cells are required for K/BxN autoimmune arthritis development (Fig. 2a, [11, 12]). However, to our surprise, SFB colonization did not increase the splenic Th17 cell number in middle-aged K/BxN mice. The smaller number of SFB-induced splenic Th17 cells is not due to decreased Th17 cell proliferation, as Ki-67, a cellular marker for proliferation, was expressed at a similar percentage in Th17 cells in both the young and middle-aged groups regardless of SFB status. The deficiency of SFB-mediated Th17 induction in middle-aged mice was not only limited at the systemic lymphoid sites. In the lung, though SFB induced Th17 cells in middle-aged mice, the total Th17 cell numbers were severely reduced compared to their young counterparts (Fig. 2b). Th17 cells are an abundant population at steady state in gut-associated tissues, particularly the small intestinal lamina propria (SI-LP) [31]. We observed that SFB-mediated Th17 cell induction was non-existent in SI-LP in middle-aged mice. As in the spleen, we also did not observe defects in Th17 proliferation in the lung or SI-LP of middle-aged mice and the percentages of their cycling Ki67^+^ Th17 cells were similar to the young group (Fig. 2b and c), and were relatively high compared to another T effector population, Tfh cells, from middle-aged mice that we examined later (Fig. 5a). Taken together, these data demonstrate that there is a defect in the middle-aged Th17 cell response to the gut microbiota SFB, though the defect is not due to a decrease in Th17 cell proliferation.

### Middle-aged autoimmune CD4^+^ T cells are defective in Th17 differentiation

**Fig. 3:**
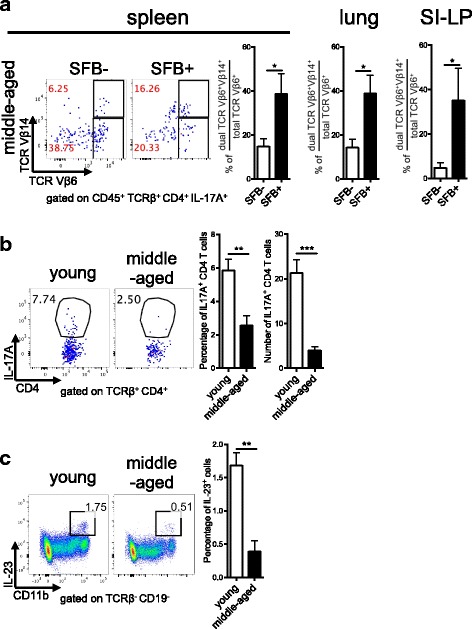
Middle-aged naïve T cells display impaired T helper 17 (Th17) cell differentiation. **a** Representative plots of T cell receptor (*TCR*) Vβ6 vs. TCR Vβ14 expression in splenic Th17 cells from segmented filamentous bacteria-negative (*SFB*–) and SFB+ middle-aged K/BxN mice. The quantitative data show the percentage of skewed Vβ14^+^Vβ6^+^ usage are calculated by 100 × [Vβ6^+^Vβ14^+^ Th17 cell percentage **⁄**total (both Vβ14^-^ and Vβ14^+^) TCR Vβ6^+^ Th17 cell percentage]. The quantitative data on TCR skewing in the lung and small intestine-lamina propria (*SI-LP*) are also shown (n = 3–6 mice in each group). **b** Fluorescence-activated cell-sorted splenic naïve CD4^+^ T cells from SFB– young and middle-aged K/BxN mice were in vitro cultured under Th17 polarization conditions for 4 days. Representative plots and quantitative data on Th17 polarization are shown (n = 4–7 mice in each group). **c** Representative plots and quantitative data on IL-23 expression in the spleen of SFB+ young and middle-aged K/BxN mice (n = 3 mice in each group)

As we did not see a defect in middle-aged Th17 cell proliferation, we next addressed whether the lower Th17 response in the middle-aged K/BxN mice is due to a defect in Th17 differentiation. In young mice, SFB-induced Th17 differentiation requires direct recognition of SFB by an SFB-specific T-cell receptor (TCR), and dominant TCR Vβ14 usage in the Th17 cells of young mice that recognize SFB peptide has been reported [27, 32]. Importantly, Th17 cells from middle-aged K/BxN did display an induction of Vβ14 usage in their endogenous TCR Vβ chain after SFB colonization in addition to the KRN TCR transgene Vβ6 in K/BxN mice (Fig. 3a). We found that SFB colonization preferentially increased the population of TCRVβ6^+^ Vβ14^+^ Th17 cells by showing a skewed percentage of KRN Th17 co-expressing Vβ6 and Vβ14 in the total TCR Vβ6 (TCR Vβ6^+^ expressing either Vβ14^+^ or Vβ14^−^) population in the spleen (Fig. 3a). We also observed the skewing of Vb14 usage on KRN autoimmune Th17 cells isolated from the lung and SI-LP (Fig. 3a). This suggests that middle-aged Th17 cells were capable of upregulating the SFB-specific Vβ14 TCR and the defect in SFB-induced Th17 cell response in middle-aged K/BxN mice is not due to lack of SFB exposure or recognition. We next tested whether there was an intrinsic defect in the ability of naïve CD4^+^ autoimmune T cells to undergo Th17 differentiation leading to the lower SFB-induced Th17 response in middle-aged mice. Naive KRN CD4^+^ T cells were sorted from young and middle-aged K/BxN mice and cultured in a Th17 polarizing environment. Despite being supplied with the optimal TCR plus co-stimulus signals with Th17 polarizing cytokines, TGF-β and IL-6, middle-aged autoimmune CD4^+^ T cells display an impaired ability to undergo Th17 differentiation (Fig. 3b). Thus, middle-aged CD4^+^ T cells have an intrinsic defect in Th17 differentiation. Finally, we also examined the T cell-extrinsic molecular mechanism that may explain the low Th17 response observed in middle-aged mice, despite having SFB+ status. When we examined a few crucial cytokines required for the development of the Th17 cell response, we found that there was decreased expression of IL-23, a critical cytokine for the maintenance of Th17 cells [33], in CD11b^+^ cells from SFB+ middle-aged mice compared to SFB+ young mice (Fig. 3c). Together, these data provide both T-cell-intrinsic and T-cell-extrinsic mechanisms explaining the Th17 defect in middle-aged K/BxN mice.

### A robust Tfh cell response leads to a strong germinal center (GC) B cell development in middle-aged K/BxN mice

**Fig. 4:**
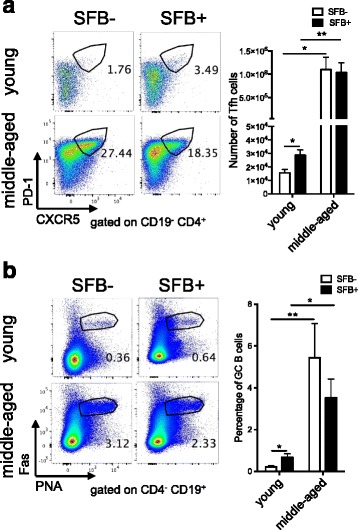
Enhanced T follicular helper (*Tfh*) and germinal center (GC) responses in middle-aged K/BxN mice. **a** Representative Tfh percentage plots and quantitative data on Tfh cell numbers in spleen from segmented filamentous bacteria-negative (*SFB*–) and SFB+ young or middle-aged K/BxN mice (n = 8–18 mice in each group). **b** Representative GC percentage plots and quantitative data on GC B cell numbers in spleen from experiments shown in **a**

We next examined Tfh cells, a key cell type involved in Ab-mediated autoimmune diseases. We compared the Tfh cell response between young and middle-aged groups in the spleen, which is a systemic lymphoid tissue and the major autoantibody production site corresponding to arthritis development [34]. SFB colonization induced a Tfh cell (defined by PD-1^+^CXCR5^+^) response in young K/BxN mice as we have reported previously (Fig. 4a, [12]). The Tfh cell response increased robustly in the middle-aged compared to the young group regardless of SFB status. Tfh cells are the key cell type involved in inducing GC responses. Because GCs play a key role in the T-cell-dependent Ab response, we compared the GC responses of SFB– and SFB+ K/BxN mice in both age groups. Our results showed a greater induction in the GC B cell (defined by Fas^+^PNA^+^) population in the spleen of SFB+ versus SFB– young controls (Fig. 4b). There was a tremendous increase in the GC B cell population in middle-aged mice regardless of SFB status. These data indicate that a strong Tfh and GC B cell response occurs with age and some specific gut microbiota such as SFB can elevate the young Tfh and GC immune compartment to mimic the increase in Tfh and GC response in middle-aged mice.

### Tfh cell proliferation and differentiation in young and middle-aged K/BxN mice

**Fig. 5:**
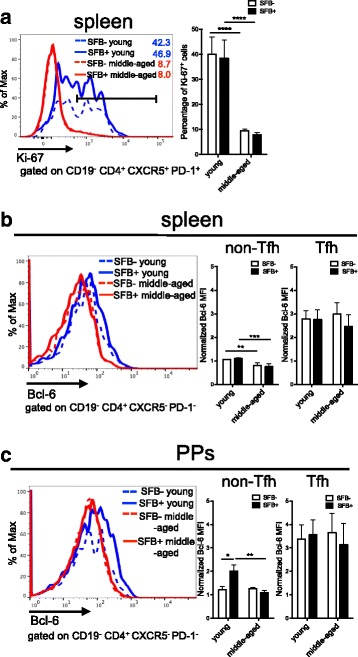
Reduced T follicular helper (*Tfh*) proliferation and differentiation in middle-aged K/BxN mice. **a** Representative histogram overlays and quantitative data on Ki-67^+^ Tfh cells in the spleen from segmented filamentous bacteria-negative (*SFB*–) and SFB+ of young or middle-aged K/BxN mice (n = 4–14 mice of each group). **b**, **c** Representative histogram overlays of Bcl-6 expression in non-Tfh cells and quantitative data on normalized Bcl-6 mean fluorescence intensity (*MFI*) in non-Tfh and Tfh cells from the spleen (**b**) and Peyer’s patches (*PPs*) (**c**) from experiments shown in **a**. Bcl-6 MFI was normalized to splenic non-Tfh cells from one SFB– young mouse within each experiment

We next asked what contributed to the robust Tfh response in the middle-aged mice. We first examined whether Tfh cells exhibit stronger proliferation with age in both SFB+ and SFB– conditions. To our surprise, there was a decrease in cycling Tfh cells as demonstrated by the lower percentage of Ki67^+^ Tfh cells in middle-aged mice regardless of SFB status (Fig. 5a). We then asked whether there was an increase of Tfh cell differentiation with age that caused the increase in the Tfh cell response in middle-aged mice. Bcl-6 is the master transcriptional regulator of Tfh cells, and promotes differentiation of non-Tfh CD4^+^ T cells into Tfh cells [16, 35]. Thus, we next examined the expression of Bcl-6 and found that there was a mild but significant drop in Bcl-6 expression in the non-Tfh cells of middle-aged compared to young mice in both the SFB– and SFB+ groups (Fig. 5b). As we have previously demonstrated that SFB specifically induce Tfh differentiation in Peyer’s patches (PPs) but not in the spleen of young K/BxN mice, we also examined Bcl-6 expression in PPs. Though SFB induced Bcl-6 upregulation in PP non-Tfh cells in the young group, there was a lack of Bcl-6 induction in the non-Tfh cells in the middle-aged group (Fig. 5c). As reported in previous studies [16, 17], once differentiated into Tfh cells, Bcl-6 was expressed at a higher level than in non-Tfh cells and there were no differences in Bcl-6 expression between the young and middle-aged groups (Fig. 5b and c). These results suggest that the increase in Tfh cell response in middle-aged K/BxN mice was not due to enhancement of Tfh cell proliferation or differentiation.

### Excessive accumulation of effector and effector memory Tfh cells in middle-aged K/BxN mice

**Fig. 6:**
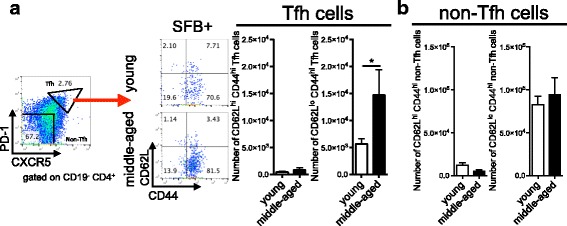
Accumulated T follicular helper (*Tfh*) cells of effector phenotype in middle-aged K/BxN mice. **a** Representative plots and quantitative data on CD62L^hi^CD44^hi^ and CD62L^lo^CD44^hi^ Tfh cell numbers in pooled spleen and lymph node cells from segmented filamentous bacteria (*SFB*+) young or middle-aged K/BxN mice (n = 4–7 in each group). **b** Quantitative data on CD62L^hi^CD44^hi^ and CD62L^lo^CD44^hi^ non-Tfh cell numbers in pooled spleen and lymph node cells from the experiment shown in **a**

Since we did not observe increased Tfh cell proliferation or differentiation in the middle-aged mice, we next addressed whether the soaring Tfh response in middle-aged mice was elicited by an accumulation of active and/or memory Tfh cells. To investigate this, we focused on SFB colonized mice, which display a strong Tfh response in both age groups, and analyzed the surface expression of CD62L and CD44 on Tfh cells. Our results revealed an increase in effector and/or effector memory-type T cells (CD62L^lo^CD44^hi^) but not central memory-type T cells (CD62L^hi^CD44^hi^) in middle-aged compared to young mice (Fig. 6a). This analysis revealed that the Tfh cells that were elevated with age were of an effector phenotype, which contrasts with non-Tfh cells, where we did not observe a difference in the effector phenotype subpopulations between the young and middle-aged groups (Fig. 6b). Thus, the significant increase in Tfh cells in middle-aged mice is contributed by the accumulation of Tfh cells of an effector phenotype.

## Discussion

Despite progress in understanding loss of immune function in Tfh cells and GC B cells with age, much less is known about age-associated changes in these cell types in an autoimmune setting. We have found that in contrast to Th17 cells, Tfh cells are heavily accumulated in middle-aged autoimmune arthritic mice. Most of the Tfh cells in middle-aged K/BxN mice are of the effector phenotype (CD62L^lo^CD44^hi^). However, we believe these CD62L^lo^CD44^hi^ Tfh cells are mostly effector memory cells and not effectors. Our rationale is that in contrast to their young counterparts, when middle-aged non-Tfh cells from PPs were exposed to SFB, a potent Tfh stimulus, they were not able to upregulate their Bcl-6 and undergo SFB-induced Tfh differentiation. Therefore, we propose that these middle-aged Tfh cells are generated at a young age while Tfh differentiation is active. They later survive as memory Tfh cells, which can readily become effector Tfh cells upon encountering self-antigen. In support of this hypothesis, we found that middle-aged Tfh cells in K/BxN mice are rather quiescent, demonstrated by their lower proliferation phenotype, which potentially helps their long-term survival [36].

The data presented here, together with previous reports, suggest that the Tfh cells accumulated over time are major contributors to autoantibody production and arthritis development in middle-aged K/BxN mice. To begin with, the autoantibody level is an important disease index in the K/BxN model. We and others have reported that arthritis can be induced simply by passively transferring anti-GPI autoantibodies or serum containing anti-GPI autoantibodies into wild-type mice [37, 38]. Our results also indicate that, compared to young mice, middle-aged mice had higher autoantibody titers, which corresponded with their increased ankle thickness (Fig. 1c). Secondly, our previous data showed that Tfh cells are required for autoantibody production in K/BxN mice by demonstrating that mice receiving CXCR5-deficient KRN T cells, which have impaired Tfh function, developed far fewer autoantibodies and less severe disease compared to those that received CXCR5-sufficient KRN T cells [12]. Third, a soaring Tfh cell population accompanied by a robust GC B cell response was found in middle-aged mice compared to the young group (Fig. 4a and b), and it has been well-established that active Tfh cells are required to maintain GC B cell populations [16, 17]. Finally, Bcl-6 is expressed at similar levels in Tfh cells in both the young and middle-aged groups (Fig. 5b and c). Because Bcl-6 is the master regulator of Tfh cells and controls the expression and function of many Tfh cell molecules [16, 17, 39], together with the robust GC B cell response in the middle-aged group, these data suggest that middle-aged Tfh cells are functionally competent. Collectively, these data suggest that the accumulated Tfh cells in middle-aged K/BxN mice are key contributors to the robust GC B cell response, which in turn leads to increased autoantibody production and arthritis development in middle-aged mice. It is worth mentioning that research focusing on aging carries unique challenges, mainly attributable to the fact that the time required to age mice makes designing gain-of-function or loss-of-function experiments with older mice much less feasible than studies involving only young subjects. However, this is an urgent topic that requires intense future focus, as age is a major risk factor for countless diseases, including many autoimmune diseases, and the world’s elderly population has been increasing at an unprecedented rate.

Our results thus could help explain the long-standing observation that with age, there is a reduction in Ab response to infection or vaccination, whereas there is an increase in autoantibody production in autoimmune patients, a phenomenon that might appear to be controversial at first sight [40]. This can be explained by an elegant study showing that despite aging mice displaying an increased Tfh population, the Tfh response to viral infections in aging mice is impaired, which is indicated by a severe reduction in the germinal center B cell response [41]. This is in sharp contrast to our results, which demonstrate that the increased Tfh population helps B cells to develop a robust GC B cell response in an autoimmune condition. Our results provide a potential mechanism to explain these seemingly paradoxical findings. We propose that during the aging process, there are reduced T effector cell activities toward new antigens. Indeed, our results show declining differentiation of Th17 and Tfh cells in response to a newly introduced antigen, SFB, in middle-aged mice. However, autoimmune diseases such as RA often have a latent phase [42], during which autoimmune Tfh cells respond to self-antigen from youth while Tfh differentiation is still active, and are accumulated with age. Because of their preferential accumulation and survival advantage over other non-Tfh cells during the aging process (Fig. 6a and b), Tfh cells eventually emerge as a significant cell type involved in the pathogenesis of RA in middle age. Recently, a population of blood circulating Tfh (cTfh) cells has been described as memory Tfh cells [43, 44]. In support of our hypothesis that memory Tfh cells could contribute to autoimmunity in middle-age, a higher frequency of circulating Tfh cells have been detected in patients with RA, and there is a positive correlation of increased cTfh cells with disease activity and autoantibody production [45]. T follicular regulatory cells (Tfr), serve as important counter-inhibitors that downregulate the Tfh cell response. It has been proposed that an increased ratio of inhibitory Tfr cells in aged mice contributes to defective Ab production in aging upon antigen immunization [46]. However, in an autoimmune setting in middle-aged mice, we did not observe representation of Tfr cells over and above Tfh cells that could have better countered the accumulated Tfh population (unpublished observations).

It has been demonstrated that there is an increase in IL-17^+^ CD4^+^ T cells in aging mice and one of the reports further demonstrated that memory Th17 cells are an important source of IL-17 production in aging mice [47, 48]. Our findings suggest that there is an overall reduction in Th17 cells in middle-aged K/BxN mice, which also displayed decreased differentiation of IL-17-producing cells from naive CD4^+^ T cells compared to the young group. The discrepancy between our results and previous mouse data could be due to the difference between middle-aged mice used in this study compared to aged mice in the former study. The other, more likely reason is the difference between using mice with an autoimmune background (K/BxN) compared to wild-type mice (B6 and CBA) used in the previous study. On that note, it is worth mentioning that similar to our findings, there were two studies in humans reporting a reduction in IL-17^+^CD4^+^ T cells in the elderly compared to the young [49, 50]. As in humans, CD4^+^ T cells from autoimmune mice such as K/BxN mice are more antigen-experienced compared to naïve wild-type mice such as B6 and CBA. It will be interesting to determine whether the antigen experience of T cells contributes to the differences in IL-17 response between our findings and previous reports.

In humans, initial evidence suggests an important role for IL-17 in the pathogenesis of several inflammatory diseases, including RA [20, 51]. However it was later discovered that the increased expression of IL-17 is not restricted to synovial tissue in patients with RA; it is also observed in patients with psoriatic arthritis and inflammatory osteoarthritis. The heterogeneous expression pattern of IL-17 in patients with RA has been proposed to be responsible for the non-responsiveness to anti-IL-17 clinical therapy in RA [52]. The K/BxN model results further suggest that the problematic maintenance of Th17 cells in middle-aged compared to young individuals may further contribute to the ineffectiveness of anti-IL-17 therapy in patients with RA, who are mostly middle-aged and older individuals. This is supported by a report showing a significant but small number of IL-17^+^ CD4^+^ T cells are detected (0.9-1.2%) in the synovial fluid and peripheral blood of patients with RA [53]. In this case, anti-IL-17 treatment may function better in younger or pre-clinical subgroups of patients.

Aging-associated alterations in gut microbiota composition have been well-documented [8, 9]. They are suggested to be caused by an age-related decline in immune function. When we colonized K/BxN mice with SFB, we observed that middle-aged K/BxN mice displayed a trend toward an increase in susceptibility to SFB colonization compared to their young counterparts. Normal gut IgA is a key immune player in reducing SFB colonization [54]. It is possible that the defect in SFB-induced Tfh differentiation in PPs indicated by the lack of SFB-mediated Bcl-6 induction in middle-aged mice could lead to reduced production of SFB-specific IgA, which results in an expansion of SFB in the gut. However, it appears that T cells in the middle-aged are less capable of responding to SFB, which can be seen at a molecular level, compared to young T cells. For example, in young K/BxN mice, we have previously reported that SFB promote non-Tfh cells differentiating into Tfh cells in PPs by upregulating Bcl-6 expression in non-Tfh cells in PPs [12]. On the other hand, SFB colonization does not upregulate Bcl-6 expression in non-Tfh cells in PPs in middle-aged K/BxN mice.

A strong Tfh response can still be observed in SFB– middle-aged K/BxN mice, suggesting that commensals other than SFB may substitute for SFB in promoting the Tfh response during the aging process, which results in a strong accumulation of Tfh cells despite the lack of SFB. In contrast, SFB is more specifically required for Th17 than Tfh responses, which is supported by data from Littman’s group [27]. Middle-aged K/BxN mice display reduced but still significant SFB-mediated Th17 induction in the lung (but not in the spleen or SI-LP), further supporting SFB-specific Th17 induction. We have previously reported that Tfh and Th17 cells can both contribute to autoantibody and arthritis development in young K/BxN mice [12]. Thus, our data from middle-aged K/BxN mice suggest that the enormous amount of Tfh cells in the middle-aged group is sufficient to induce a robust GC B cell response that promotes severe arthritis with less help from Th17 cells. The reduced but still significant induction of Th17 cells (at least in the lung) in SFB colonized mice may partly contribute to the higher anti-GPI titer in SFB+ middle-aged K/BxN mice. Although this did not contribute to visible pathological differences in the mice, which could be due to the limitations of detection of disease symptoms and severity in mice, the differential auto-Ab titer may still contribute to different degrees of disease manifestation among patients. Using TCR Tg K/BxN mice in our study allowed us to significantly control the TCR repertoire over the aging process. However, it is a well-recognized phenomenon that incomplete allelic exclusion of TCRs can lead to dual TCR expression on T cells, not only in TCR Tg mice, but also in wild-type mice and humans [55, 56]. Furthermore, dual TCR expression has been reported to increase with aging [22]. Thus, it will be very interesting to determine whether microbiota contribute to dual TCR expression during the aging process, and how this may impact autoimmune development.

Our data also suggest that certain microbiota, such as SFB, may render young hosts prone to an overactive immune phenotype, as evidenced by the young K/BxN mice colonized with SFB displaying stronger disease and immune phenotypes, such as increased Tfh and GC responses, that are reminiscent of, though still much lower than, those in middle-aged mice. Interestingly, patients with RA have a phenotype of premature immune aging (immunosenescence) including telomere shortening and contraction of the T cell repertoire [57]. Further investigation is required to determine whether immunosenescence in RA could be contributed to by the human equivalent of microbiota species similar to mouse SFB, with strong host immunomodulation effects.

## Conclusions

The K/BxN mice allow us to systemically examine the age-associated and gut microbiota- associated impact on pathological T effector cells, Tfh and Th17 cells, in the development of autoantibody-mediated autoimmune arthritis. Our results show that despite both Tfh and Th17 cells having been reported to participate in the pathogenesis of autoimmune arthritis, there is a clear age-associated accumulation of Tfh but not Th17 cells in the middle-aged group compared to their young counterparts. SFB colonization, a potent stimulus for inducing Tfh differentiation, fails to promote Tfh differentiation in the middle-aged group, which suggests that most of the middle-aged Tfh cells with an effector phenotype (CD62L^lo^CD44^hi^) are Tfh effector memory cells induced at an earlier age. Our results also indicate that exposure to certain benign commensals may cause the young host to display an overactive immune system similar to the middle-aged group. Targeting the survival of Tfh cells and colonization of immunomodulatory commensals may offer immune-based and/or microbe-based therapies in RA.

## Abbreviations

Ab: Antibody
Ag: Antigen
B6: C57BL/6
cTfh: Circulating T follicular helper
DMEM: Dulbecco’s modified Eagle’s medium
EDTA: Ethylenediaminetetraacetic acid
ELISA: Enzyme-linked immunosorbent assay
FBS: Fetal bovine serum
FCS: Fetal calf serum
FITC: Fluorescein isothiocyanate
GC: Germinal center
GPI: Glucose-6-phosphate isomerase
H&E: Hematoxylin and eosin
iBALT: Inducible bronchus-associated lymphoid tissue
IL-17A/IL-17: Interleukin-17A
K/BxN: F1 mice of KRN/B6 x NOD
mAbs: Monoclonal antibodies
MFI: Mean fluorescence intensity
PBS: Phosphate-buffered saline
PNA: Peanut agglutinin
PPs: Peyer’s patches
RA: Rheumatoid arthritis
SFB: Segmented filamentous bacteria
SI-LP: Small intestine-lamina propria
TCR: T cell receptor
Tfh: T follicular helper
Tfr: T follicular regulatory cells
Tg: Transgenic
TGF: Transforming growth factor
Th17: T helper 17
